# Activation of G protein‐coupled parathyroid hormone receptors in rat incisor odontoblasts promotes mineralization via cyclic adenosine monophosphate, not Ca^2+^ signalling: In vitro study

**DOI:** 10.1111/iej.14280

**Published:** 2025-07-11

**Authors:** Natsuki Saito, Takehito Ouchi, Maki Kimura, Ryuya Kurashima, Yoshiyuki Shibukawa

**Affiliations:** ^1^ Department of Physiology Tokyo Dental College Chiyoda‐ku Tokyo Japan; ^2^ Department of Dental Anesthesiology Tokyo Dental College Chiyoda‐ku Tokyo Japan

**Keywords:** cyclic adenosine monophosphate (cAMP), mineralization, odontoblasts, parathyroid hormone (PTH)

## Abstract

**Aim:**

Parathyroid hormone (PTH) and its Gα_s_‐coupled receptors, PTH receptor, mediate odontoblast differentiation; however, the detailed intracellular adenylyl cyclase‐mediated signalling pathway mediated by the PTH–PTH receptor axis remains to be elucidated. Therefore, we measured the intracellular levels of cyclic adenosine monophosphate (cAMP) in living single odontoblasts.

**Methodology:**

We obtained acutely isolated odontoblasts from newborn Wistar rats and analysed the mineralization ability by Alizarin red staining. Intracellular‐free Ca^2+^ concentration was measured using a fluorescent Ca^2+^ indicator, whereas intracellular cAMP levels were examined by a mNeon Green–based cAMP sensor.

**Results:**

Granulated PTH was detected in the vascular area of the dental pulp periphery. Application of the non‐selective PTH receptor agonist DPC AJ1951 increased cAMP levels in odontoblasts. This increase was significantly inhibited by the non‐selective PTH receptor antagonist 4185‐v and the adenylyl cyclase inhibitor SQ 22536. However, applying the non‐selective PTH receptor agonist DPC AJ1951 did not increase the intracellular Ca^2+^ concentration without extracellular Ca^2+^. In mineralization assays, PTH promoted mineralization by odontoblasts. The mineralization was inhibited by SQ 22536 and 4185‐v but not by the phospholipase C inhibitor U73122.

**Conclusion:**

Thus, the present study suggests that PTH from the bloodstream functionally activates the Gα_s_‐coupled PTH receptor in odontoblasts, which plays an essential role in dentinogenesis.

## INTRODUCTION

Parathyroid hormone (PTH) is an endocrine hormone secreted by the parathyroid glands that regulates serum extracellular calcium and phosphate concentrations, affecting bone, kidney and intensive metabolism, and thus bone homeostasis (Lyu et al., 2021). PTH plays a role in bone remodelling by targeting skeletal stem cells, bone marrow stromal cells, osteoprogenitors, osteocytes and osteoclasts (Rhee et al., 2011; Wein & Kronenberg, 2018). PTH exerts its actions through the PTH/PTH‐related peptide (PTHrP) receptor, also known as PTH1 receptor (PTH1R), a G protein‐coupled receptor. PTH1R couples to multiple G proteins, including Gα_s_ and Gα_q_/_11_, mainly expressed in bone and kidney to regulate Ca^2+^ homeostasis. PTH can also bind to and activate PTH2R; however, it is now known that PTH2R is primarily involved in the action of the tuberoinfundibular peptide of 39 residues (TIP39) specifically (Bastepe et al., 2017). TIP39 and PTH2R are abundantly expressed in the amygdala, parabrachial nuclei, locus coeruleus and nucleus of the solitary tract. The TIP39–PTH2R axis forms a neuromodulatory system (Faber et al., 2007).

Heterotrimeric Gα_s_ protein‐coupled receptor (GPCR)‐regulated adenylyl cyclase signal transduction pathways produce intracellular cyclic adenosine monophosphate (cAMP) signalling pathway, while activation of Gα_q_‐coupled metabotropic receptor induces intracellular Ca^2+^ signalling pathway via phospholipase C (PLC) signalling cascade, resulting in the inositol 1,4,5‐triphosphate (IP_3_) production which activates Ca^2+^ release from intracellular Ca^2+^store. Both lead to various highly specialised cellular functions mediated by their downstream signalling cascades (Chaudhary & Kim, 2021). In addition, its downstream signalling enables further biological effects (Chaudhary & Kim, 2021).

In dental pulp cells, bone morphogenetic protein‐2, a crucial regulator of mineralization, is regulated by the cAMP and extracellular signal‐regulated kinase 1/2 pathways (Tada et al., 2011). Dental pulp stem cells also express PTH1R, and its activation plays important roles in odontogenic differentiation as well as endodontic regeneration therapy by participating in dental repair processes (Lyu et al., 2021). PTH1R contributes to dentine regeneration by promoting odontoblast differentiation after severe dental tissue damage (Zhao et al., 2021) and dental pulp inflammation (Marigo et al., 2010). Dynamic changes in the intracellular cAMP level activated by the PTH receptor, which involves odontoblast function, remain to be clarified; however, excessive internal Ca^2+^ induced by intracellular Ca^2+^ mobilization pathways is extruded via the Na^+^–Ca^2+^ exchanger and/or plasma membrane Ca^2+^‐ATPase (PMCA) in odontoblasts to regulate intracellular Ca^2+^ levels (Kimura et al., 2021). The Ca^2+^ extrusion mechanism via Na^+^–Ca^2+^ exchanger and PMCA at the distal membrane of odontoblasts may serve as a directional Ca^2+^ transport pathway from the circulation to the dentine mineralizing front by transporting accumulated intracellular Ca^2+^ resulting from accelerated Ca^2+^ signalling events activated by physiological/pathophysiological dental pulp responses or stimuli external to the dentine (Kimura et al., 2021; Tsumura et al., 2010).

PTH receptor coupled with Gα_s_ and/or Gα_q_ protein and their second messenger signals are mediated by intracellular cAMP and/or Ca^2+^, respectively, and may function in dentine mineralization. We, thus, aimed to investigate the intracellular signalling pathway activated by the PTH–PTH receptor axis coupled with both Gα_s_ and Gα_q_ proteins and their effects on dentine mineralization.

## MATERIALS AND METHODS

**FIGURE 1:**
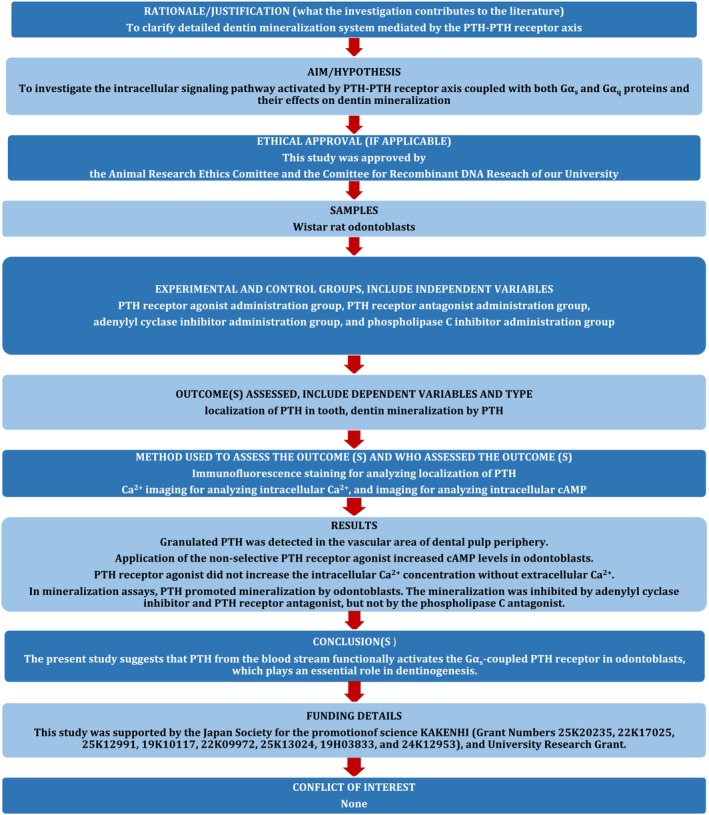
PRILE 2021 flowchart. From: Nagendrababu et al. (2021). For further details visit: http://pride‐endodonticguidelines.org/prile.

The manuscript of this study has been written according to Preferred Reporting Items for Laboratory Studies in Endodontology (PRILE) 2021 guidelines (Figure 1).

### Solutions and reagents

A solution containing 136 mM NaCl, 5 mM KCl, 0.5 mM MgCl_2_, 10 mM 2‐[4‐(2‐hydroxyethyl)‐1‐piperazinyl] ethanesulfonic acid, 10 mM glucose, and 12 mM NaHCO_3_ (pH 7.4 with tris [hydroxymethyl] aminomethane) with (standard ECS) or without 2.5 mM CaCl_2_ (Ca^2+^‐free ECS) was used as an extracellular solution (ECS). The potent nonselective PTH receptor agonist DPC AJ1951 (Carter et al., 2007), adenylyl cyclase inhibitor SQ 22536 (Liu et al., 2020; Tang et al., 2020) and PLC inhibitor U73122 (Shi et al., 2008) were obtained from R&D Systems, Inc. (Minneapolis, MN, USA). The pharmacological potent nonselective antagonist of the PTH receptor, 4185‐v (Horiuchi et al., 1983), was obtained from PEPTIDE INSTITUTE, Inc. (Ibaragi, Osaka, Japan). Stock solutions of SQ 22536 were prepared in dimethyl sulfoxide. Stock solutions for others were prepared using ultrapure water (Millipore, Burlington, MA, USA). The stock solutions were diluted with standard ECS or Ca^2+^‐free ECS to an appropriate concentration before use. We used a gravity‐fed perfusion system during cAMP/Ca^2+^ recordings (Warner Instruments, Holliston, MA, USA) to apply pharmacological agents diluted in the ECS or to exchange ECS. All other reagents were purchased from Sigma Aldrich (St. Louis, MO, USA).

### Preparation of frozen sections

The mandibles were dissected and collected from newborn Wistar rats (aged 4–8 days). Decapitation was performed under isoflurane (3%) and pentobarbital sodium anaesthesia (25 mg/kg, administered via intraperitoneal injection). Both male and female rats were used in this study to reduce the number of euthanized rats. The samples were then fixed in 4% paraformaldehyde (PFA) (FUJIFILM Wako Pure Chemical Co., Osaka, Japan) in phosphate‐buffered saline (PBS) (14190144, Thermo Fisher Scientific, Waltham, MA, USA; 045‐29795, FUJIFILM Wako Pure Chemical Co., Osaka, Japan) overnight, transferred to 10% and 30% sucrose (FUJIFILM Wako Pure Chemical Co., Osaka, Japan)/PBS solution for 3–5 h, and then embedded in OCT compound (4583, Sakura Finetek Japan, Tokyo, Japan). Frozen tissue sections were cut into 10‐μm‐thick slices using a Leica CM1950 cryostat (Leica Biosystems, Wetzlar, Germany).

### Dental pulp slice preparation

Dental pulp slices were obtained from newborn Wistar rats (aged 4–8 days) (Kimura et al., 2018; Shibukawa et al., 2015; Shibukawa & Suzuki, 1997, 2003). Decapitation was performed under isoflurane (3%) and pentobarbital sodium anaesthesia (25 mg/kg, administered via intraperitoneal injection). Both male and female rats were used in this study to reduce the number of euthanized rats. The mandibles were dissected, and the hemi mandibles embedded in alginate impression material were sectioned transversely through the incisor at a thickness of 500 μm using a standard vibration tissue slicer (ZERO‐1; Dosaka EM, Kyoto. Japan). The mandibular sections were sliced so that the dentine and enamel were directly visible between the bone tissue and dental pulp. The surrounding impression material, bone tissue, enamel and dentine were carefully removed, and the remaining dental pulp slices were obtained. Pulp slices were treated with ECS containing 0.17% collagenase and 0.03% trypsin at 37°C for 30 min. For measuring the intracellular cAMP level and intracellular‐free Ca^2+^ concentration as well as for mineralization assay, the enzymatically treated dental pulp slices were plated in culture dishes (81156‐400, ibidi, Bayern, Germany), bathed in alpha‐minimum essential medium (Life Technologies Co., Grand Island, NY, USA) containing 10% foetal bovine serum (Life Technologies Co.), 5% horse serum, 1% amphotericin B and 1% penicillin–streptomycin (Life Technologies Co.) and maintained at 37°C in a 5% CO_2_ incubator for 24–40 h. Bright field images of cultured dental pulp slices were observed under a microscope, Leica DMI3000 B (Leica Microsystems, Wetzlar, Germany).

### Haematoxylin–eosin staining and immunofluorescence staining

Frozen sections were stained with haematoxylin–eosin under the standard protocol. Stained samples were mounted on glass slides and enclosed by using an encapsulant (FX00100, Matsunami, Osaka, Japan). Frozen sections and dental pulp slices (maintained in the culture medium for 20–40 h) were also used in immunofluorescence analysis described below. Samples were fixed with 4% PFA (FUJIFILM Wako Pure Chemical Co.) and washed with PBS (Life Technologies Co.). After incubation with 0.1%–0.3% Triton X‐100 (Sigma Aldrich, St. Louis, MO, USA), blocking reagent (Nacalai Tesque, Kyoto, Japan) was applied at room temperature. The following primary antibodies were applied for 3–4 h at room temperature or overnight at 4°C: mouse monoclonal anti‐dentine sialophosphoprotein (DSPP) (Santa Cruz Biotechnology, Inc., Dallas, TX, USA; sc‐73632, 1:200), mouse monoclonal anti‐CD31 (Santa Cruz Biotechnology; sc‐376764, 1:200), rabbit monoclonal anti‐PTH (ABclonal, Woburn, MA, USA; A9704, 1:200), mouse monoclonal anti‐PTH/PTHrP receptor (PTH1R) (Santa Cruz Biotechnology, Inc.; sc‐12722, 1:200), rabbit polyclonal anti‐PTH1R (ABclonal; A1744, 1:200), rabbit polyclonal anti‐heterotrimeric G‐protein α‐subunit Gα_s_ (Gnas) (ABclonal; A5546, 1:200) and mouse monoclonal anti‐heterotrimeric G‐protein α‐subunit Gα_q_ (Gnaq) (Santa Cruz Biotechnology; sc‐136181, 1:200). For the negative control, we used mouse polyclonal IgG isotype control antibody (abcam, Cambridge, UK; ab37355) and rabbit polyclonal IgG isotype control antibody (proteintech, Rosemont, IL, USA; 30000‐0‐AP). We used the following secondary antibodies: Alexa Fluor® 568 donkey anti‐mouse (Life Technologies Co., #A10037) and Alexa Fluor® 488 donkey anti‐rabbit (Life Technologies Co., #A21206). Secondary antibodies were applied for 1 h at room temperature. Stained samples were mounted using a mounting reagent containing 4,6‐diamidino‐2‐phenylindole (DAPI) (abcam; ab104139). Stained samples were observed under a microscope (BZ‐X710, BZ‐X800; Keyence, Osaka, Japan).

### Intracellular cAMP level assays in living odontoblasts

To monitor cAMP dynamics in odontoblasts, we changed the culture medium for dental pulp slices to one containing 0.4% Na‐Butyrate and 16.7% BacMam sensor (green upward cAMP difference detector in situ [cADDis]; Montana Molecular, Bozeman, MT, USA) after 24–36 h of isolation (i.e. after 24–36 h for primary culture). The odontoblasts were then incubated in the medium at 37°C for 20–40 h and washed with standard ECS. BacMam sensor‐transfected odontoblasts were observed using a microscope (IX73, Evident Co., Tokyo, Japan), which was equipped with an intensified charge‐coupled device camera system, an excitation wavelength selector and an HCImage system (Ver. 4.3.1, Hamamatsu Photonics, Shizuoka, Japan). cADDis fluorescence emission (F_506_) was measured at 517 nm in response to an excitation wavelength of 506 nm. The intracellular cAMP level was represented as the fluorescence ratio (F/F_0_ cAMP) of the F506 value (F) to its resting value (F_0_). The intracellular cAMP level was analysed in the designated measurement field (region of interest, ROI), where cADDis‐transfected and ‐expressed odontoblasts were located in the peripheral area of dental pulp slice preparation. All experiments were performed at room temperature (28°C).

### Measurements of intracellular‐free Ca^2+^concentration in living odontoblasts

We loaded fura‐2 acetoxymethyl ester (10 μM) (Dojindo, Kumamoto, Japan) (with pluronic acid F‐127 (0.1% (w/v)); Life Technologies Co.) into primary cultured odontoblasts in standard ECS or Ca^2+^‐free ECS for 90 min at 37°C. We then rinsed the cultured odontoblasts with fresh standard ECS or Ca^2+^‐free ECS and placed them on a microscope stage (IX73). We measured fura‐2 fluorescence emission at 510 nm by alternating excitation wavelengths of 340 nm (F_340_) and 380 nm (F_380_) (HCImage software). The intracellular Ca^2+^ level was analysed in ROI, where fura‐2‐loaded odontoblasts were located. The software controls an intensified charge‐coupled device camera system (Hamamatsu Photonics) and a selector for excitation wavelength. We measured intracellula‐free Ca^2+^ concentration ([Ca^2+^]_i_) using the fluorescence ratio of F_340_ to F_380_ (R_F340/F380_) at two excitation wavelengths. The changes in [Ca^2+^]_i_ were described in F/F_0_ Ca^2+^ units; the R_F340/F380_ value (F) was normalized to the resting value (F_0_). We performed all the experiments at room temperature (28°C).

### Mineralization assay

Isolated odontoblasts existed at the outermost layers in the dental pulp slices, which were grown for 20–40 h in basal medium and then transferred to mineralization medium (adding 10 mM β‐glycerophosphate and 50 μg/mL ascorbic acid to basal medium) for growth at 37°C in 5% CO_2_. To determine the effects of PTH activity on mineralization, odontoblasts were cultured in mineralization medium without (as control) or with the non‐selective PTH receptor agonist, DPC AJ1951 (50 nM), as well as with the non‐selective PTH receptor antagonist, 4185‐v (50 nM), AC inhibitor SQ 22536 (0.1 μM) or PLC inhibitor U73122 (1 μM) for 7 days. During the 7‐day culture period, the mineralization medium with or without pharmacological agents was changed twice weekly. To detect the deposition of mineralized nodules, cells were subjected to Alizarin red staining, and mineralization efficiencies were measured using a microscope (BZ‐X710, BZ‐X800; Keyence). We measured the whole Alizarin red‐stained superficial area in sliced dental pulp (*S*). We also measured the Alizarin red‐stained superficial area of inner dental pulp (*S*_0_) indicating that the mineralized superficial area driven by odontoblasts was represented as (*S − S*_0_) to evaluate the mineralized superficial area by odontoblasts. To normalize differences in the superficial area of inner dental pulp and to evaluate mineralizing efficiency by odontoblasts as increased superficial area, we calculated the *S*/*S*_0_ unit (Saito et al., 2022).

### Statistical analysis

Data are expressed as the mean ± SE or SD of the mean of *N* observations, where *N* represents the number of experiments or cells tested. Non‐parametric statistical significance was determined using the Friedman test and Mann–Whitney *U* test with Dunn's post‐hoc test to analyse intracellular cAMP levels, intracellular Ca^2+^ concentrations and mineralized areas in rat odontoblasts (Figures 4, 5, 6). Statistical significance was set at *p* < .05. Statistical analyses were performed using GraphPad Prism 8.0 (GraphPad Software, La Jolla, CA, United States). The data were analysed using Origin 8.5 (OriginLab Corporation, Northampton, MA, USA).

## RESULTS

### PTH1R expression in DSPP‐positive rat odontoblasts in vivo

**FIGURE 2:**
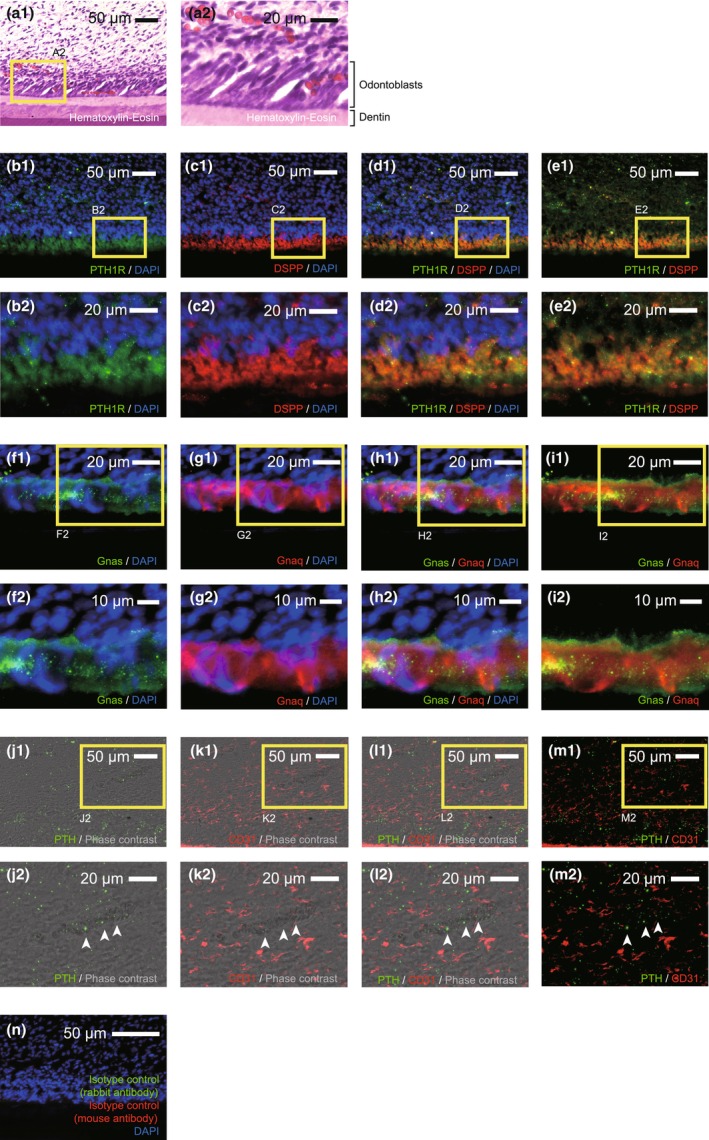
Expression of PTH1R in the rat incisor odontoblasts in vivo. Haematoxylin–Eosin images show tall columnar shaped odontoblasts in rat incisors of the mandibular sagittal bone sections (a1 and a2). The magnified image (a2) shows the outlined area by the yellow rectangle in (a1). Immunofluorescence staining images show the dentine‐pulp border of rat mandibular incisors (b1–n). The magnified images show the outlined areas by the yellow rectangles in b1 for b2, c1 for c2, d1 for d2, e1 for e2, f1 for f2, g1 for g2, h1 for h2, i1 for i2, j1 for j2, k1 for k2, l1 for l2, m1 for m2. Odontoblasts in the peripheral region of the mandibular incisor dental pulp were immunopositive for PTH1R (green in b1, b2, d1, d2, e1 and e2), mature odontoblast marker DSPP (red in c1, c2, d1, d2, e1 and e2), Gnas, which encodes Gα_s_ protein (green in f1, f2, h1, h2, i1 and i2), and Gnaq, which encodes Gα_q_ protein (red in g1, g2, h1, h2, i1 and i2). In the dental pulp beneath the odontoblast layer, PTH expression (green in j1, j2, l1, l2, m1 and m2) was detected in the vascular lumen (white arrowheads in j2, k2, l2 and m2) within blood vessel wall indicated by endothelial cell marker CD31 (red in k1, k2, l1, l2, m1 and m2) positive cells. No fluorescence expression was detected in negative control experiments by using species specific isotype controls (n). The nuclei (DAPI staining) are shown in blue. Representative pictures from (a1) to (n) were selected from the results in each experiment (*N* = 4). Scale bars: 50 μm in a1, b1, c1, d1, e1, j1, k1, l1, m1 and n; 20 μm in a2, b2, c2, d2, e2, f1, g1, h1, i1, j2, k2, l2 and m2; 10 μm f2, g2, h2 and i2.

To evaluate the PTH1R expression in dental pulp, we performed immunostaining. In the outermost layer, we observed tall columnar cells in haematoxylin–eosin‐stained samples (Figure 2a1,a2). These tall columnar cells were immunopositive for PTH1R (green in Figure 2b1,b2,d1,d2,e1,e2), DSPP (red in Figure 2c1,c2,d1,d2,e1,e2). Odontoblasts were also immunopositive for Gnas, which encodes Gα_s_ protein (green in Figure 2f1,f2,h1,h2,i1,i2), and Gnaq, which encodes Gα_q_ protein (red in Figure 2g1,g2,h1,h2,i1,i2). These results suggested that PTH1R‐positive odontoblasts are regulated by Gα_s_‐ and/or Gα_q_‐protein‐coupled receptors in their functions. Beneath the odontoblast layer, granulated PTH (green in Figure 2j1,j2,l1,l2,m1,m2) was detected around the CD31‐positive area (red in Figure 2k1,k2,l1,l2,m1,m2). Our results indicated that PTH may be coupled with functions in PTH1R‐positive odontoblasts. Immunostaining using isotype controls revealed that no fluorescence detections were observed (Figure 2n).

### Identification of PTH1R‐positive odontoblasts after dental pulp slice preparations

**FIGURE 3:**
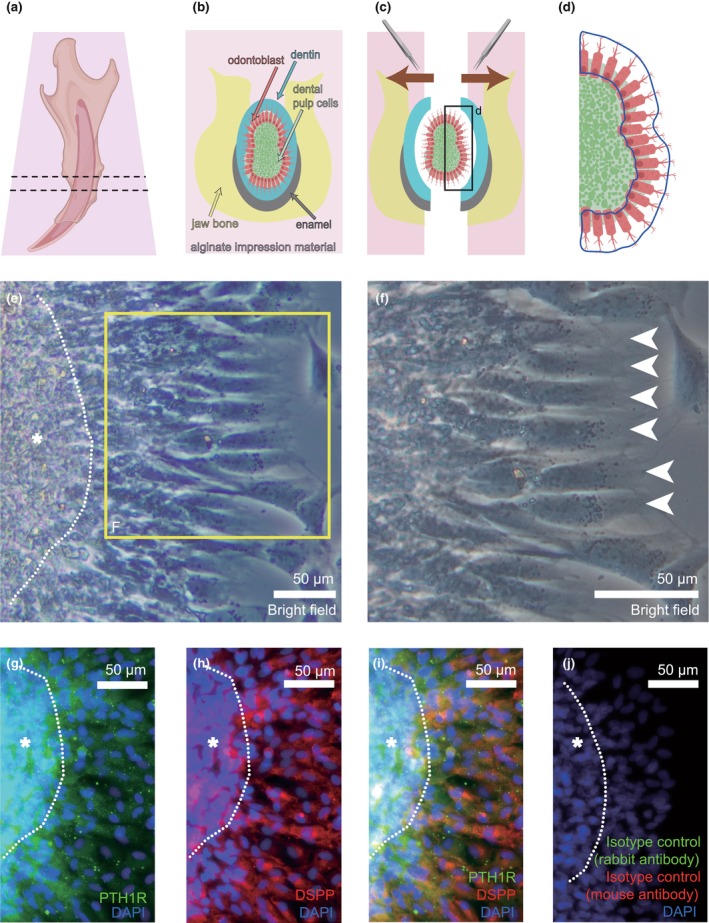
PTH1R expression in DSPP‐positive rat odontoblasts in sliced dental pulp. Schematic images show the procedure to prepare acute isolation of rat odontoblasts in the incisor dental pulp slices (a–c). Rat mandibles were embedded in alginate impression materials (a). A mandible slice, including dental pulp surrounded by the impression material, was prepared as coronal sections. In the preparation of the mandible slice, tissues including jaw bone (yellow), enamel (grey), dentine (cyan), odontoblasts (red) and dental pulp cells (green) were appropriately maintained in their anatomical positions (b). Under the stereoscopic microscope, the surrounding impression material, jaw bone, enamel and dentine were carefully removed outwards by forceps as shown by brown arrows. The remaining dental pulp slice including odontoblasts was subsequently obtained (c). We evaluated intracellular cAMP level, intracellular‐free Ca^2+^ concentration and mineralization level in cells located at the outermost layers of the slice as odontoblasts outlined by the blue line (d). The magnified drawing (d) shows the outlined area by the black rectangle in (c). Bright field images show odontoblast layers (e and f). Inner dental pulp cells (indicated by an asterisk) are outlined by a white dotted line (e). The magnified image (f) shows the outlined area by the yellow rectangle in (e). Odontoblasts (indicated by white arrowheads in f) were located at the periphery of the dental pulp cells in the slices (e). The presence of nuclei was observed at the distal side within odontoblasts (f). Representative pictures in (e and f) were selected from results (*N* = 3). Odontoblasts at the outermost layers in dental pulp slices were immunopositive for PTH1R (green in g and i) and mature odontoblast marker, DSPP (red in h and i). No fluorescence expression was detected in negative control experiments by using species‐specific isotype controls (j). Dental pulp cells (indicated by asterisks) are outlined by white dotted lines (g–j). The nuclei (DAPI staining) are shown in blue. Representative pictures in (g–j) were selected from the results in each experiment (*N* = 3). Scale bars: 50 μm in (e–j).

We next prepared dental pulp slices to obtain acutely isolated odontoblasts (see Materials and Methods) and to evaluate the functions of the PTH–PTH1R axis in the odontoblasts (Figure 3a–d). From whole mandibular slices (Figure 3a,b), we removed alginate impression materials and tissues (Figure 3c) surrounding odontoblasts, including bone, enamel and dentine, as well as connective tissues, and subsequently obtained dental pulp slices. In the slices, we could observe odontoblasts at the outermost layers of dental pulp. Thus, we achieved primary cultured odontoblasts in the dental pulp slices with appropriate anatomical positions (Figure 3d). We could observe nuclei at the distal side within tall columnar‐shaped odontoblasts at the periphery of the dental pulp slices (Figure 3e,f). Primary cultured odontoblasts showed immunopositivity for PTH1R (green in Figure 3g,i) and DSPP (red in Figure 3h,i), demonstrating that the odontoblasts in sliceddental pulp maintained the odontoblast marker protein, as previously reported (Tsumura et al., 2012). Immunostaining using isotype controls revealed that no fluorescence detections were observed (Figure 3j).

### DPC AJ1951, a non‐selective PTH receptor agonist, increased adenylyl cyclase (AC)‐dependent intracellular cAMP levels

**FIGURE 4:**
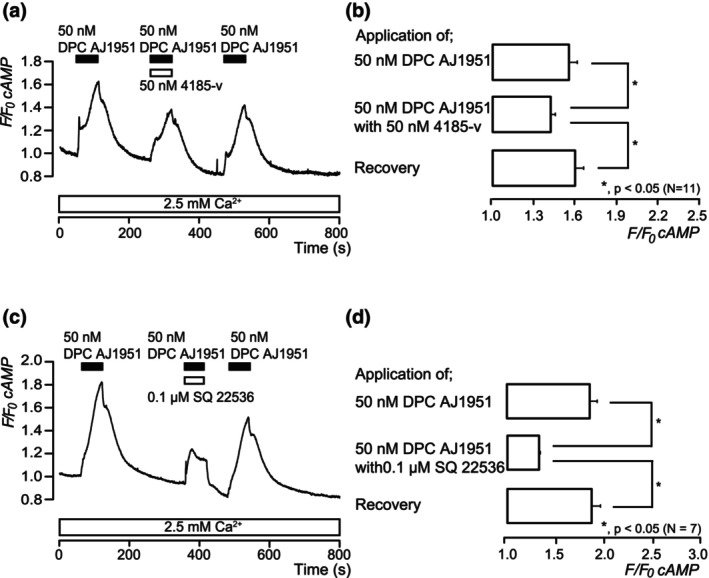
Increases in intracellular cAMP level by PTH receptor activation in odontoblasts. Representative traces of the transient increases in intracellular cAMP level by application of 50 nM DPC AJ1951 with or without 50 nM 4185‐v (a) or 0.1 μM SQ 22536 (c) in the presence of extracellular Ca^2+^ (2.5 mM) (white boxes at the bottom). Black boxes indicate the periods of DPC AJ1951 application to the extracellular solution. White boxes at the top indicate the time of addition of 50 nM 4185‐v (a) or 0.1 μM SQ 22536 (c) to the extracellular solution. (b, d) Summary bar graphs show DPC AJ1951‐induced intracellular cAMP level increases in the absence (upper columns) or presence (middle columns) of 50 nM 4185‐v (b) or 0.1 μM SQ 22536 (d) in the presence of extracellular Ca^2+^ (2.5 mM). Each recovery effect (lower columns in b, d) shows the reversible effect after application of 4185‐v (b) or SQ 22536 (d). Each bar denotes the mean ± SE. Numbers in parentheses show the number of experiments. Asterisks represent statistically significant differences between columns (shown by solid lines): *p < .05.

In the presence of extracellular Ca^2+^ (2.5 mM), application of 50 nM of DPC AJ1951, a non‐selective PTH receptor agonist, to odontoblasts showed rapid and transient increases in intracellular cAMP level, reaching peak values of 1.56 ± 0.06 (*N* = 11; Figure 4a,b) and 1.86 ± 0.77 (*N* = 7; Figure 4c,d) in *F/F*_0_ cAMP units, followed by a rapid decay to near baseline levels (*F/F*_0_ cAMP = 1). DPC AJ1951‐induced—non‐selective PTH receptor agonist—cAMP level increases were significantly and reversibly inhibited by 50 nM 4185‐v, a non‐selective PTH receptor antagonist, to 1.43 ± 0.34 (*N* = 11; Figure 4a,b) and 0.1 μM SQ 22536, a selective AC inhibitor, to 1.33 ± 0.01 (*N* = 7; Figure 4c,d) in *F/F*_0_ cAMP units, reversibly.

### DPC AJ1951 did not increase intracellular‐free Ca^2+^ concentration

**FIGURE 5:**
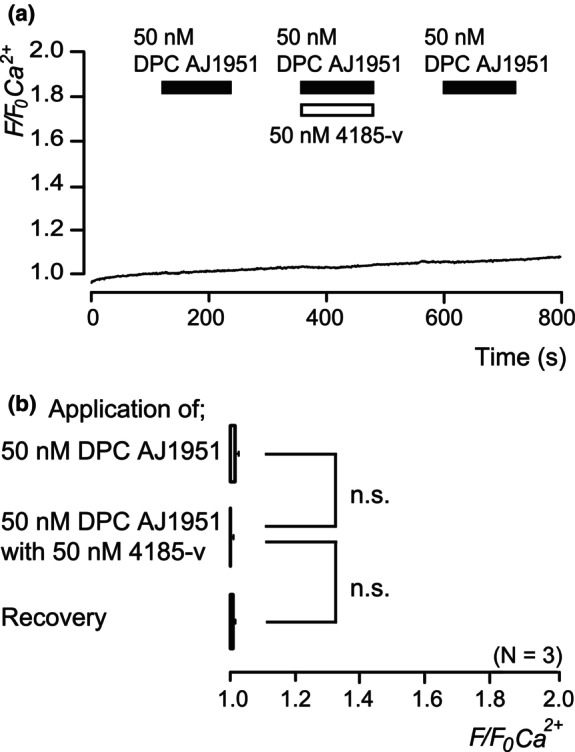
PTH receptor activation did not contribute to the intracellular Ca^2+^ releasing pathway. Representative trace of increase in the intracellular‐free Ca^2+^ concentration ([Ca^2+^]_i_) by application of 50 nM DPC AJ1951, without or with 50 nM 4185‐v in the absence of extracellular Ca^2+^ (a). Black boxes indicate the time of application of DPC AJ1951 to the extracellular solution. The white box at the top indicates the time of addition of 4185‐v. Summary bar graphs show nothing changes in [Ca^2+^]_i_ by DPC AJ1951 application without (upper column) or with 50 nM 4185‐v (middle column) in the absence of extracellular Ca^2+^ (0 mM) (b). Recovery effect (lower column) is shown. Each bar denotes the mean ± SE. Numbers in parentheses indicate the number of experiments. N.s. denote no statistically significant differences between columns (shown by solid lines).

In the absence of extracellular Ca^2+^, applying 50 nM DPC AJ1951, a non‐selective PTH receptor agonist, did not increase the intracellular Ca^2+^ concentration in *F/F*_0_ Ca^2+^ units (Figure 5a,b), suggesting that PTH receptor activation did not contribute to the Gα_q_ protein‐mediated PLC cascade and subsequent IP_3_‐induced Ca^2+^ release.

### PTH receptor signalling, via Gα_s_ protein‐mediated AC cascade, regulates dentine mineralization

**FIGURE 6:**
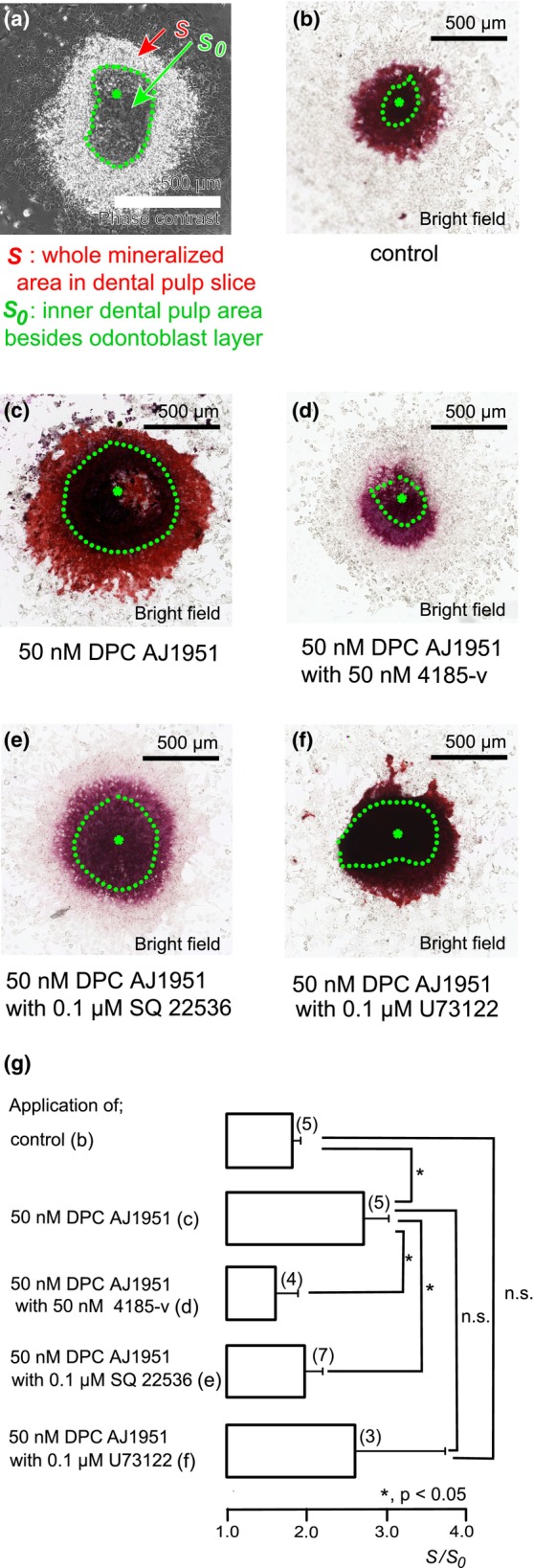
PTH receptor‐induced signalling enhanced mineralization by odontoblast. Mineralization area driven by odontoblasts was analysed and evaluated based on a diagram shown by phase contrast picture which was obtained after mineralization induction (a). The mineralized area driven by the whole Alizarin red‐stained superficial area in sliced dental pulp (*S*) was analysed. In addition, the Alizarin red‐stained superficial area of inner dental pulp (*S*_0_) was also analysed. Dental pulp cells are shown as a green asterisk surrounded by a green dotted circle (a). To normalize differences in the superficial area of inner dental pulp and to evaluate mineralizing efficiency by odontoblasts as increased superficial area, we calculated the ratio, *S*/*S*_0_ unit. Rat incisor dental pulp slices including odontoblasts were cultured for 7 days in a mineralization medium without pharmacological intervention (b) or with 50 nM DPC AJ1951 (c), 50 nM DPC AJ1951 and 50 nM 4185‐v (d), 50 nM DPC AJ1951 and 0.1 μM SQ 22536 (e), 50 nM DPC AJ1951 and 0.1 μM U73122 (f) at pH 7.4. After mineralization induction of dental pulp slices, samples were stained by using Alizarin red solution in order to detect mineralized nodules. Green dotted lines (b–f) indicate the borders between odontoblasts and inner dental pulp (shown by green asterisks) according to (a). (g) Mineralized area by odontoblasts was statistically analysed. The estimated mineralization levels were 1.82 ± 0.10 *S*/*S*_0_ in the absence of PTH receptor modifiers (as controls), 2.71 ± 0.31 *S*/*S*_0_ with 50 nM DPC AJ1951, 1.61 ± 0.28 *S*/*S*_0_ with 50 nM DPC AJ1951 and 50 nM 4185‐v, 1.97 ± 0.23 *S*/*S*_0_ with 50 nM DPC AJ1951 and 0.1 μM SQ 22536, and 2.60 ± 1.13 *S*/*S*_0_ with 50 nM DPC AJ1951 and 0.1 μM U73122. Each column denotes the mean ± SE of each experiment. Numbers in parentheses show the number of experiments. Statistically significant differences between columns (indicated by solid lines) are indicated by asterisks. *p < .05.

We investigated the effects of PTH1R activity on mineralization efficacy in primary cultured odontoblasts in dental pulp slices. Alizarin red staining in odontoblast layers was determined to be indicative of the mineralized areas (see Materials and Methods and Figure 6a) represented as *S*/*S*_0_ units. After 7 days of induction, it was revealed that mineralized nodules were detected in odontoblast layers (Figure 6b,g). Applying 50 nM of DPC AJ1951 to odontoblasts significantly increased their mineralization levels (Figure 6c,g) compared with those in the control group. Increases in mineralization by 50 nM of DPC AJ1951 were significantly inhibited by the addition of 50 nM 4185‐v (Figure 6d,g) or the addition of 0.1 μM SQ 22536 (Figure 6e,g). However, when we applied 0.1 μM of PLC inhibitor, U73122, in addition to 50 nM of DPC AJ1951 to the odontoblasts, their mineralization levels were similar to those by the application of DPC AJ1951 only (Figure 6f,g).

## DISCUSSION

Odontoblasts in dental pulp slices were identified based on their expression of DSPP, a mature odontoblast marker (Krivanek et al., 2020; Won et al., 2017). We showed expression of PTH in the location just beneath odontoblasts and areas of CD31‐positive endothelial cells, suggesting that PTH was capable of being released from blood vessels to the odontoblast in dental pulp. DSPP‐positive rat odontoblasts also express PTH1R, which responds to PTH and PTHrP ligands (Kobayashi et al., 2023; Kronenberg, 2006). The Gα_s_ protein‐coupled PTH receptor activation regulates the AC signal transduction pathway to produce cAMP. In the present study, we recorded cAMP levels from the single cell, to avoid gap junctional communication comprising connexin family members between odontoblasts. Note that gap junctional communication allows intercellular communication via cAMP.

Activation of the PTH1R leads to stimulating Gα_s_ and/or Gα_q_ family heterotrimeric G proteins (Kronenberg, 2006). Genetic analyses demonstrated that Gα_s_ activation mediates the action of PTHrP to keep chondrocytes proliferating, while Gα_q_ activation opposes this action (Kronenberg, 2006). In odontoblasts, activation of metabotropic ADP receptors, Gα_q_‐coupled receptors, established intercellular odontoblast–odontoblast communication (Sato et al., 2015; Shibukawa et al., 2015) via extracellular ADP. In addition, odontoblasts also express Gα_q_‐coupled receptors (Shibukawa & Suzuki, 2003). Gα_q_ activates PLC, which produces diacylglycerol and IP_3_ from phosphatidylinositol 4,5‐bisphosphate (PIP_2_); diacylglycerol diffuses along the plasma membrane, and IP_3_ elevates the intracellular Ca^2+^ concentration by releasing Ca^2+^ from the endoplasmic reticulum, as Ca^2+^ store (Chaudhary & Kim, 2021). This IP_3_‐induced Ca^2+^ release occurs in either the presence or the absence of extracellular Ca^2+^. In our study, we could not observe any significant increase in DPC AJ1951‐induced intracellular Ca^2+^ concentration in the absence of extracellular Ca^2+^, suggesting that the PTH receptor does not contribute to activate PLC via Gα_q_ protein, leading to IP_3_‐induced Ca^2+^ release. Therefore, PTH activated AC via Gα_s_‐coupled PTH receptors in odontoblasts.

Calcium and inorganic phosphate metabolism are controlled by the orchestration of several key organs. PTH has been identified as a crucial mediator of bone formation and resorption, releasing calcium and phosphate from the matrix into the bloodstream (Lyu et al., 2021). Additionally, activation of PTH1R, which is expressed in both the adjacent dental mesenchyme and the alveolar bone, is an essential signal in the eruption pathway (Philbrick et al., 1998). PTH1R activation also mediates the later mesenchymal–epithelial interactions that are necessary for tooth maturation (Calvi et al., 2004).

In the present study, we could observe enhancement of PTH receptor‐ and AC‐mediated mineralization by odontoblasts. PTH receptor‐mediated mineralization was independent of Gα_q_‐coupled PLC activity. We, thus, propose that intracellular cAMP may regulate Ca^2+^ influx pathway to induce dentinogenesis in developmental and/or pathological settings in odontoblasts by extruding Ca^2+^ to the mineralizing front via Na^+^‐Ca^2+^ exchanger and/or plasma membrane Ca^2+^‐ATPase. AC activation might be capable of eliciting Ca^2+^ influx via calcium homeostasis regulator1 (CALHM1) (our personal communication by YS). Further studies will be needed to clarify the PTH and PTH receptor axis‐dependent Ca^2+^ influx pathway.

It has been reported that the odontogenic differentiation of dental pulp stem cells is regulated by PTH and PTH1R in an autocrine manner (Bhandi et al., 2021). However, we could observe PTH in the blood vessel located just beneath the odontoblast layer. PTH released from the vessels in the dental pulp is capable of accelerating dentinogenesis.

## CONCLUSION

We concluded that PTH activated Gα_s_‐coupled PTH receptors, but not Gα_q_‐coupled ones, and increased intracellular cAMP levels by activating AC activity in odontoblasts. The axis between PTH released from blood vessels and PTH receptor activation in odontoblasts plays essential roles in dentinogenesis.

## AUTHOR CONTRIBUTIONS

**Natsuki Saito:** Contributed to conception, design, acquisition and interpretation and drafted and critically revised the manuscript. **Takehito Ouchi:** Contributed to conception, design, acquisition and interpretation and drafted and critically revised the manuscript. **Maki Kimura:** Contributed to conception, design, acquisition and interpretation and drafted and critically revised the manuscript. **Ryuya Kurashima:** Contributed to acquisition and drafted. **Yoshiyuki Shibukawa:** Contributed to the conception, design, acquisition and interpretation and drafted and critically revised the manuscript.

## FUNDING INFORMATION

This study was supported by the Japan Society for the Promotion of Science KAKENHI [Grant Numbers 25K20235 (NS), 22K17025 (TO), 25K12991 (TO), 19K10117 (MK), 22K09972 (MK), 25K13024 (MK), 19H03833 (YS), and 24K12953 (YS)], the Tokyo Dental College Research Branding Project (Multidisciplinary Research Center for Jaw Disease (MRCJD): Achieving Longevity and Sustainability by Comprehensive Reconstruction of Oral and Maxillofacial functions), the Tokyo Dental College Research Grant (Well‐being Project) and the Tokyo Dental College Dean's Encouragement Research Grant.

## CONFLICT OF INTEREST STATEMENT

The authors declare that they have no competing financial interests or personal relationships that could have influenced the work reported in this study.

## ETHICS STATEMENT

This study was approved by the Animal Research Ethics Committee and the Committee for Recombinant DNA Research of Tokyo Dental College (numbers 230301, 240301 and DNA 1805, 2304). All animals were treated in accordance with the Guiding Principles for the Care and Use of Animals in the Physiological Sciences, approved by the Council of the Physiological Society of Japan and the American Physiological Society.

## Data Availability

All data for this study are available from the corresponding author upon request.
